# Molecular basis of anticoagulant and anticomplement activity of the tick salivary protein Salp14 and its homologs

**DOI:** 10.1016/j.jbc.2021.100865

**Published:** 2021-06-09

**Authors:** Stepan S. Denisov, Johannes H. Ippel, Elisabetta Castoldi, Ben J. Mans, Tilman M. Hackeng, Ingrid Dijkgraaf

**Affiliations:** 1 Department of Biochemistry, Cardiovascular Research Institute Maastricht (CARIM), University of Maastricht, Maastricht, The Netherlands; 2 Epidemiology, Parasites and Vectors, Agricultural Research Council-Onderstepoort Veterinary Institute, Onderstepoort, South Africa; 3 Department of Life and Consumer Sciences, University of South Africa, Pretoria, South Africa; 4 Department of Veterinary Tropical Diseases, University of Pretoria, Pretoria, South Africa

**Keywords:** coagulation factor, complement system, NMR, protein structure, parasite, ticks, lectin pathway, anticoagulants, BSA, bovine serum albumin, BSAP1, BaSO4-adsorbing protein 1, EGF, epidermal growth factor, FV, factor V, FVIIa, factor VIIa, FXa, factor Xa, HSQC, heteronuclear single quantum coherence, MASP, MBL-associated serine protease, MBL, mannan-binding lectin, SPPS, solid-phase peptide synthesis, TF, tissue factor, TFPIα, tissue factor pathway inhibitor alpha, TSLPI, tick salivary lectin pathway inhibitor

## Abstract

During feeding, a tick's mouthpart penetrates the host's skin and damages tissues and small blood vessels, triggering the extrinsic coagulation and lectin complement pathways. To elude these defense mechanisms, ticks secrete multiple anticoagulant proteins and complement system inhibitors in their saliva. Here, we characterized the inhibitory activities of the homologous tick salivary proteins tick salivary lectin pathway inhibitor, Salp14, and Salp9Pac from *Ixodesscapularis* in the coagulation cascade and the lectin complement pathway. All three proteins inhibited binding of mannan-binding lectin to the polysaccharide mannan, preventing the activation of the lectin complement pathway. In contrast, only Salp14 showed an appreciable effect on coagulation by prolonging the lag time of thrombin generation. We found that the anticoagulant properties of Salp14 are governed by its basic tail region, which resembles the C terminus of tissue factor pathway inhibitor alpha and blocks the assembly and/or activity of the prothrombinase complex in the same way. Moreover, the Salp14 protein tail contributes to the inhibition of the lectin complement pathway *via* interaction with mannan binding lectin–associated serine proteases. Furthermore, we identified BaSO_4_-adsorbing protein 1 isolated from the tick *Ornithodoros savignyi* as a distant homolog of tick salivary lectin pathway inhibitor/Salp14 proteins and showed that it inhibits the lectin complement pathway but not coagulation. The structure of BaSO_4_-adsorbing protein 1, solved here using NMR spectroscopy, indicated that this protein adopts a noncanonical epidermal growth factor domain–like structural fold, the first such report for tick salivary proteins. These data support a mechanism by which tick saliva proteins simultaneously inhibit both the host coagulation cascade and the lectin complement pathway.

Ticks (*Ixodida*) are blood-sucking ectoparasites counting circa 900 species, which are widely distributed across the globe (1). They present a threat for animal husbandry and humans, transmitting more than a dozen of serious diseases, such as Lyme disease, typhus, and tick-borne meningoencephalitis (2). Although ticks predominantly populate areas with warm and humid climate, their geographic range limit is expanding because of climate change, which will most likely dramatically increase pressures on public health and economy in the future (3). In that light, scientific research on ticks and their biomolecular adaptive mechanisms would be beneficial for the development of potential vaccines and therapeutics.

One of the crucial adaptations to the parasitic way of life that ticks acquired during evolution is an enormous arsenal of bioactive compounds in their saliva (4). Among their weaponry are such diverse compounds as anticoagulants (5), chemokine-binding proteins (6), platelet aggregation (7, 8), and complement system inhibitors (9, 10). The latter attract particular attention as the tick salivary lectin pathway inhibitor (TSLPI) of *Ixodes* *scapularis* has been shown to not only inhibit the lectin complement pathway but also facilitate the transmission of the Lyme disease agent *Borrelia burgdorferi* (9). Interestingly, the tick factor Xa (FXa) inhibitor Salp14 (11) and the fibrinolysis modulator Ixonnexin (5) share 81% and 83% sequence identity with TSLPI. Taking into account that the coagulation cascade and complement system are evolutionary deeply interconnected (12), we were intrigued by the possibility that proteins from the TSLPI/Salp14 family may target both systems.

In the present work, we have identified a distant homolog of the TSLPI/Salp14 proteins—BaSO_4_-adsorbing protein 1 (BSAP1) isolated from *Ornithodoros savignyi*. Furthermore, we have studied the activity of selected TSLPI/Salp14 proteins and BSAP1 in the complement system and coagulation cascade. Although all proteins act as lectin pathway inhibitors, those with a lysine-rich C terminus in addition possess anticoagulant activity similar to the human natural anticoagulant tissue factor (TF) pathway inhibitor alpha (TFPIα) C terminus. Moreover, structure elucidation of BSAP1 using NMR spectroscopy showed that the protein core region adopts a noncanonical epidermal growth factor (EGF)–like domain fold, which is a novel motif for tick salivary proteins.

## Results

### Homology search

**Figure 1:**
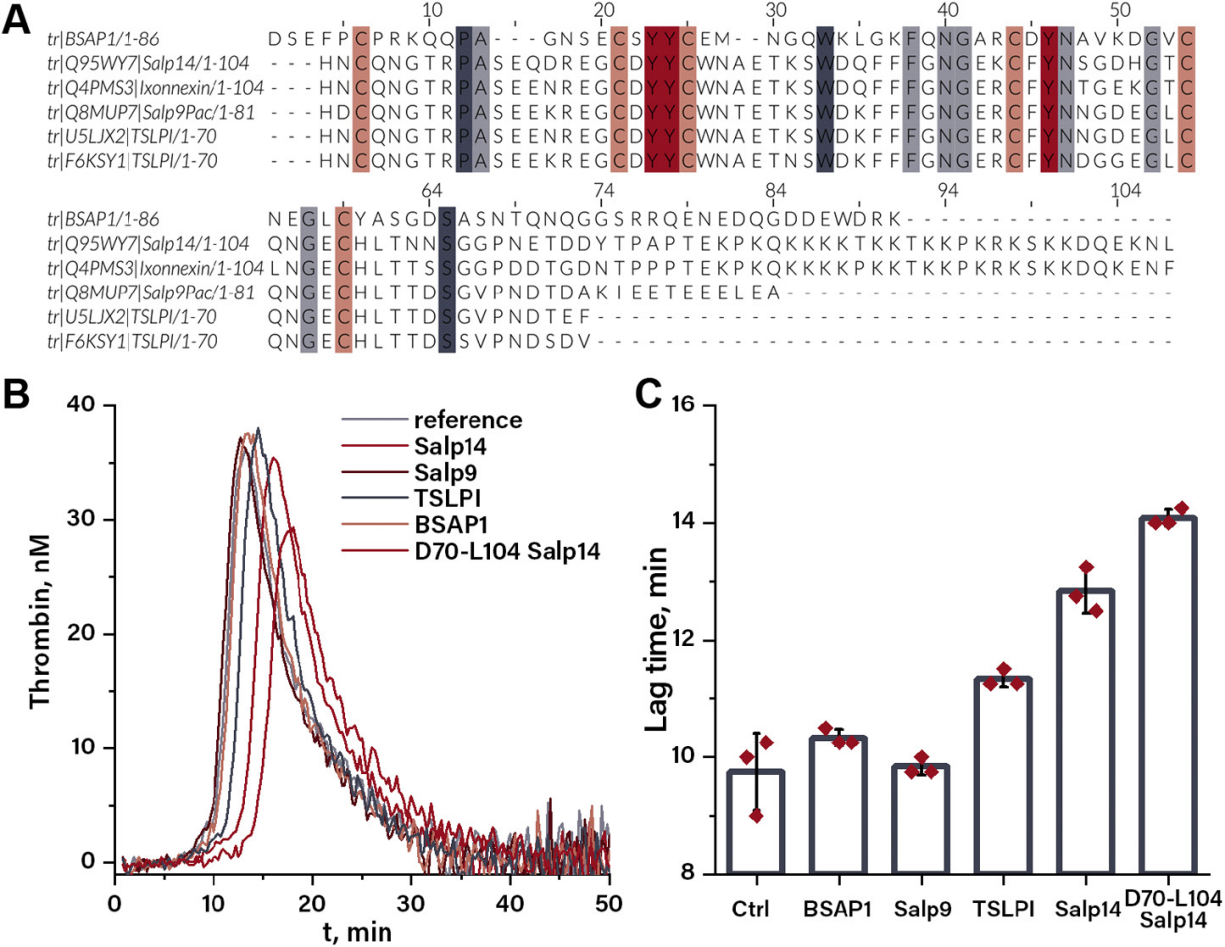
**Anticoagulant activity of TSLPI/Salp14 proteins.***A*, sequence alignment of multiple TSLPI/Salp14 proteins. *B*, thrombin generation curves at 10 μM of inhibitor. Each curve is an average of three independent measurements. *C*, lag time of thrombin generation in the absence and presence of 10 μM tick proteins. Values are averaged from three independent thrombin generation curves, and error bars indicate SD. TSLPI, tick salivary lectin pathway inhibitor.

To date, the following characterized proteins could be assigned to the TSLPI/Salp14 family: TSLPI (Uniprot accession code: U5LJX2), Salp14 (Uniprot accession code: Q95WY7), Salp9Pac (Uniprot accession code: Q8MUP7), Ixonnexin (Uniprot accession code: Q4PMS3) from *I. scapularis*, and TSLPI (Uniprot accession code: F6KSY1) from *I*. *ricinus* (5, 9, 13). Whereas these proteins share the highly conserved N-terminal region, with sequence identity varying from 82 to 94%, their C-terminal region shows no clear consensus. Salp14 and Ixonnexin have long positively charged C termini rich in polylysine stretches, whereas Salp9pac embodies a short negatively charged C-terminal region, and both TSLPI variants lack an extended C terminus (Fig. 1*A*). Alongside with these proteins, homology search resulted in several dozens of uncharacterized sequences deposited in the protein bank as part of tick salivary gland transcriptomes.

Although further homology search using the Blastp algorithm yielded no characterized homologs, we hypothesized that BSAP1 isolated from the tick *O. savignyi* (14) could be a member of the TSLPI/Salp14 family. Full-length BSAP1 is composed of 86 amino acids including six cysteine residues (15) and showed relatively low sequence identity ranging from 23 to 33% with Salp14 and TSLPI (*I. ricinus*), respectively. However, it shares a similar pattern of cysteine residues and multiple fully conserved residues among all selected proteins (P12, A13, Y20, Y21, W29, F33, N35, G36, Y41, N42, G47, G52, and S60). The C-terminal region of BSAP1 is composed predominantly of charged residues and has no homology with other TSLPI/Salp14 proteins. Considering that BSAP1 is found in soft ticks, whereas the other proteins are from hard ticks, BSAP1 could be a distant homolog of the Salp14/TSLPI protein family.

To test this hypothesis, BSAP1 and *I. scapularis* proteins, TSLPI, Salp14, and Salp9Pac, were expressed in *Escherichia coli* for further study (Table S1). All expressed proteins were obtained with a retained N-terminal methionine and then refolded under oxidative conditions (Fig. S1). Oxidative refolding resulted in a 6 Da decrease of molecular mass, indicating the formation of three disulfide bonds. Purification of refolded proteins using HPLC yielded ∼2 to 6 mg of protein per 1 l of bacterial culture.

### Effects of tick proteins/peptides on blood coagulation

The effect of tick proteins on blood coagulation was assessed by following thrombin generation in normal human plasma triggered with 2 pM TF in the absence and presence of 10 μM tick proteins. In the absence of tick proteins, thrombin generation had a lag time of 9.8 ± 0.5 min and a peak height of 35.9 ± 0.9 nM in normal pooled human plasma (Fig. 1*B*). None of the tick proteins affected the peak height of thrombin generation. BSAP1 and Salp9Pac also did not significantly affect the lag time (10.3 ± 0.1 and 9.8 ± 0.1 min, respectively), whereas addition of TSLPI caused a slight prolongation to 11.3 ± 0.1 min (Fig. 1*B*). Salp14 showed the most pronounced anticoagulant activity, increasing the lag time up to 12.8 ± 0.3 min, without affecting the peak height (35.5 ± 1.4 nM). As Salp14 is the only protein among the ones tested that embodies a positively charged C terminus, one could hypothesize that this region and not the protein core is the source of anticoagulant activity. To test this hypothesis, the D70-L104 region of Salp14 was synthesized using Boc-based solid-phase peptide synthesis (SPPS) (Fig. S2 and Table S2) and tested in the thrombin generation assay. Addition of D70-L104 Salp14 resulted in prolongation of the lag time up to 14.0 ± 0.1 min, which is comparable with the effect of the full-length protein (Fig. 1*C*).

**Figure 2:**
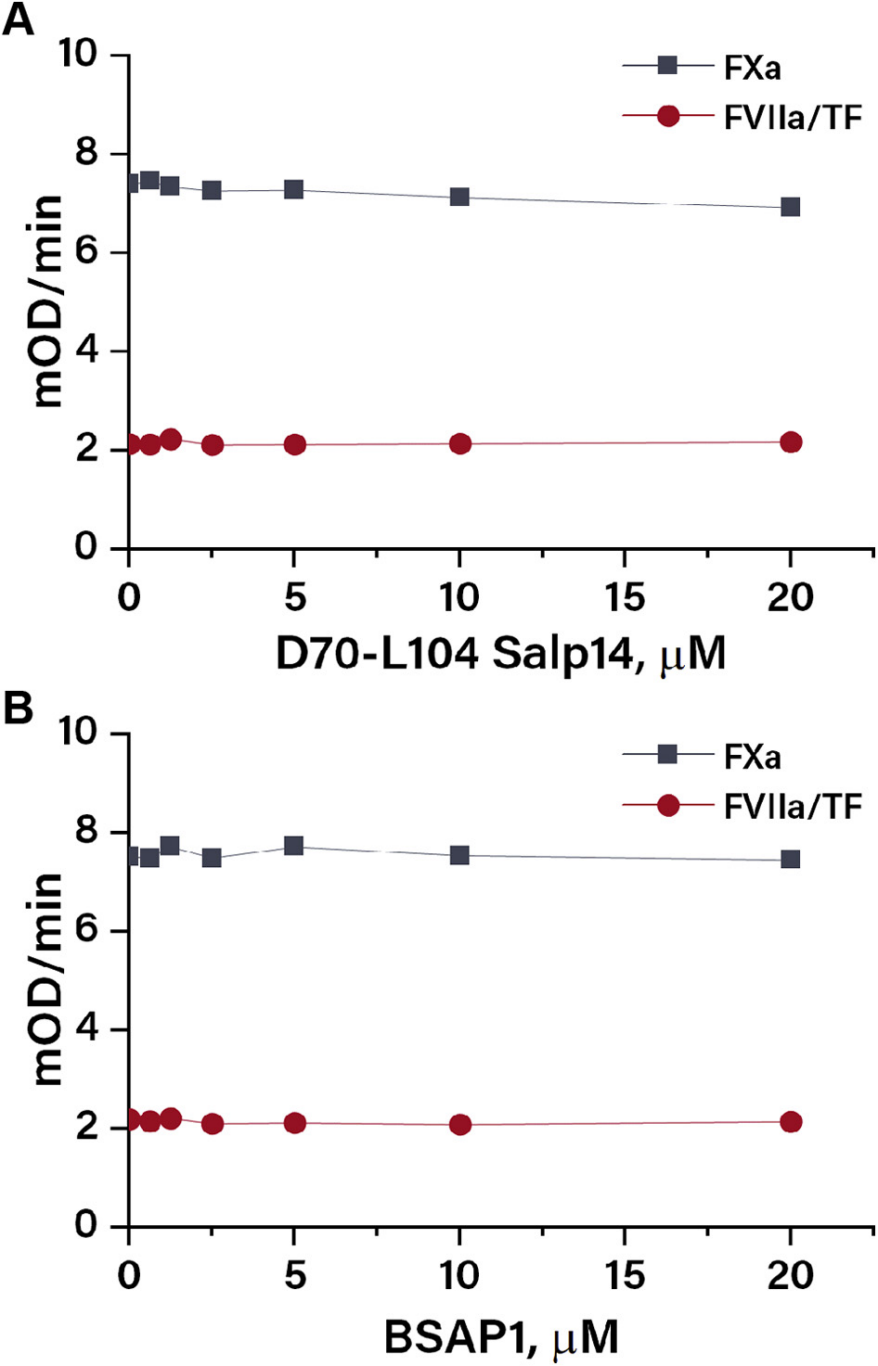
**Serine proteases inhibitory activity.** Initial slopes of absorbance curves obtained in TF/FVIIa and FXa chromogenic assays at different concentrations of D70-L104 Salp14 (*A*) and BSAP1 (*B*). BSAP1, BaSO_4_-adsorbing protein 1; FVIIa, factor VIIa; FXa, factor Xa; TF, tissue factor.

To shed light on the molecular mechanism of the anticoagulant activity of D70-L104 Salp14, its influence on individual enzymes participating in thrombin generation was studied. The ability to prolong the lag time of thrombin generation indicated inhibition of the initiation phase, in which the TF/factor VIIa (FVIIa) complex activates FX, which in turn activates factor V (FV) leading to the formation of the prothrombinase (FXa/FVa) complex. Chromogenic assays were used to study potential inhibition of TF/FVIIa and FXa activity. D70-L104 Salp14 up to a concentration of 20 μM had no effect on TF/FVIIa or FXa amidolytic activities (Fig. 2*A*), thereby failing to provide an explanation for the prolongation of the lag time of thrombin generation by this peptide. Moreover, as BSAP1 was initially described as an extrinsic pathway inhibitor (14), it was titrated in TF/FVIIa and FXa chromogenic assays as well, but similar to D70-L104 Salp14, no inhibitory activity was observed (Fig. 2*B*).

**Figure 3:**
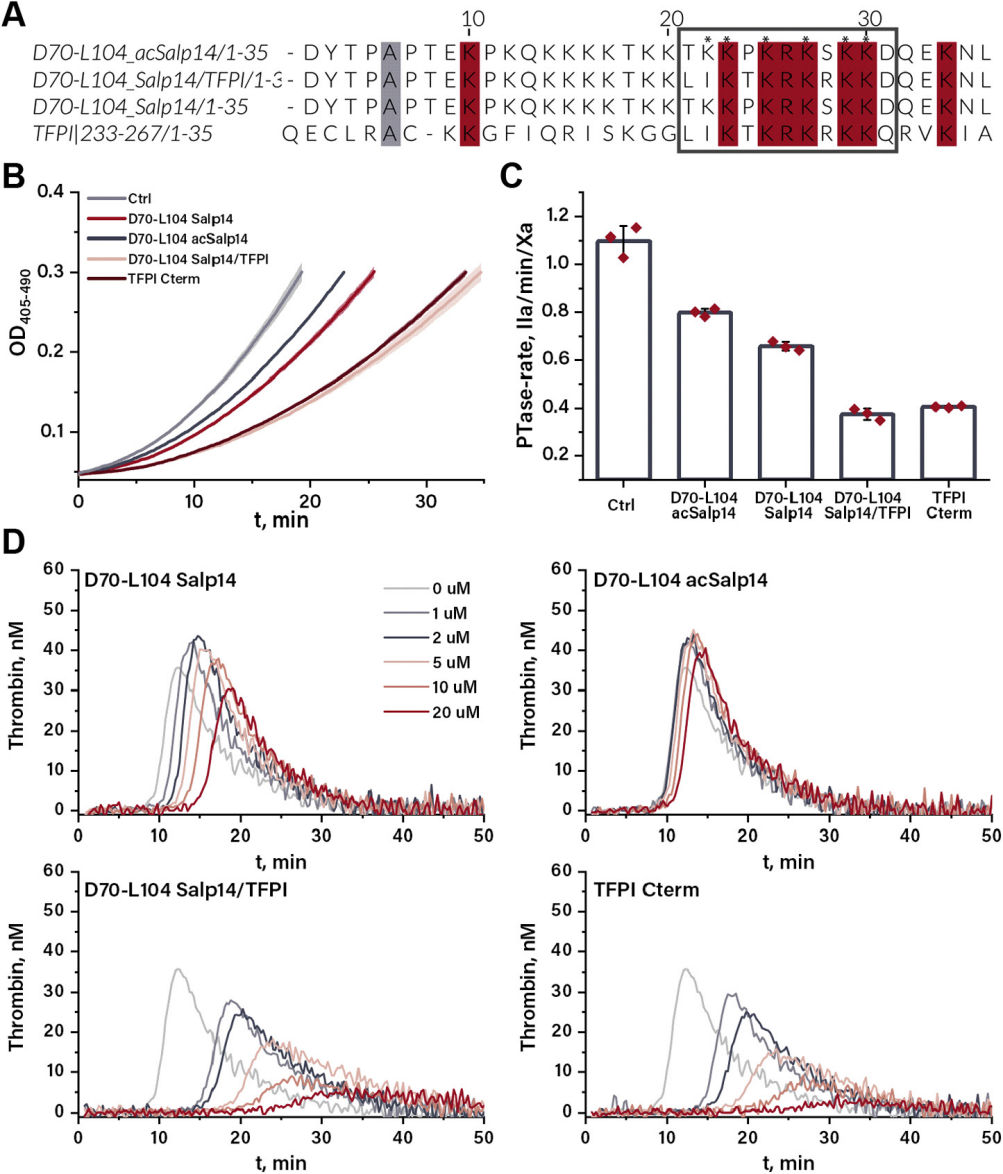
**Anticoagulant activity of D70-L104 Salp14 variants.***A*, sequence alignment of D70-L104 Salp14 variants and the C-terminal region of TFPIα. The basic region of TFPIα that is crucial for the FV(a) binding is shown in the *gray box*. Conserved residues are colored, acetylated lysines of D70-L104 acSalp14 are indicated by an ∗. *B*, absorbance traces obtained in the FV(a) inhibition assay using 1/1000 diluted pooled human plasma in the absence and presence of 100 nM inhibitor. Each curve is averaged from three measurements, and SD is shown by *shaded areas* under the curves. *C*, prothrombinase rates obtained from the parabolic fits of the absorbance tracings shown in panel *B*. The bars indicate mean values, and the error bars indicate SD. *D*, thrombin generation curves of normal human plasma at different concentration of D70-L104 Salp14 variants and TFPIα C-terminus. Each curve is averaged from two independent measurements. FV, factor V; TFPIα, tissue factor pathway inhibitor alpha.

It has been demonstrated that the basic region of the TFPIα C terminus can bind to the exposed acidic region of FV (16), thereby inhibiting both full FV activation (17) and the activity of early prothrombinase complexes that contain partially activated forms of FV (16). D70-L104 Salp14 embodies a stretch of basic residues ^89^TKKPKRKSKK^98^ closely resembling the TFPIα C-terminal residues ^252^LIKTKRKRKK^261^, which are crucial for FV binding (16) (Fig. 3*A*). Therefore, we hypothesized that the positively charged region of D70-L104 Salp14 could act in the same manner as the TFPIα C terminus and tested this hypothesis using an assay previously developed to probe the susceptibility of FV activation/activity to inhibition by the TFPIα C terminus (18). Addition of 100 nM D70-L104 Salp14 caused a 40% decrease in prothrombinase activity compared with the no-peptide reference (Fig. 3, *B* and *C*), indicating that D70-L104 Salp14 indeed interferes with FV activation and/or early prothrombinase activity.

To further elucidate the role of these basic residues, two novel peptides based on D70-L104 Salp14 were designed. In the first peptide, all six lysines in the designated ^89^TKKPKRKSKK^98^ region were acetylated to remove their positive charges, whereas in the second one, four residues were mutated to obtain the TFPIα-like sequence ^89^LIKTKRKRKK^98^. Peptides were produced with Boc-based SPPS and named D70-L104 acSalp14 and D70-L104 Salp14/TFPI, respectively (Table S2 and Fig. S2). When tested in the FV inhibition assay, 100 nM of D70-L104 Salp14/TFPI was a more potent inhibitor compared with D70-L104 Salp14, resulting in ∼30% residual prothrombinase activity. This value is comparable with the effect caused by addition of 100 nM TFPIα C terminus (Fig. 3, *B* and *C*). The acetylated variant D70-L104 acSalp14 was the least active, decreasing prothrombinase activity to ∼70% of the value observed in the absence of an inhibitor. To assess the relevance of the basic region of D70-L104 Salp14 for anticoagulant activity, its three variants alongside with the TFPIα C terminus were also tested in the thrombin generation assay (Fig. 3*D*). D70-L104 acSalp14 showed no significant influence on the lag time and peak height compared with the native sequence. In contrast, titration of D70-L104 Salp14/TFPI and the TFPIα C terminus effectively prolonged lag time and decreased the peak height of thrombin generation curves in a dose-dependent manner.

### Effects of tick proteins on complement activation

**Figure 4:**
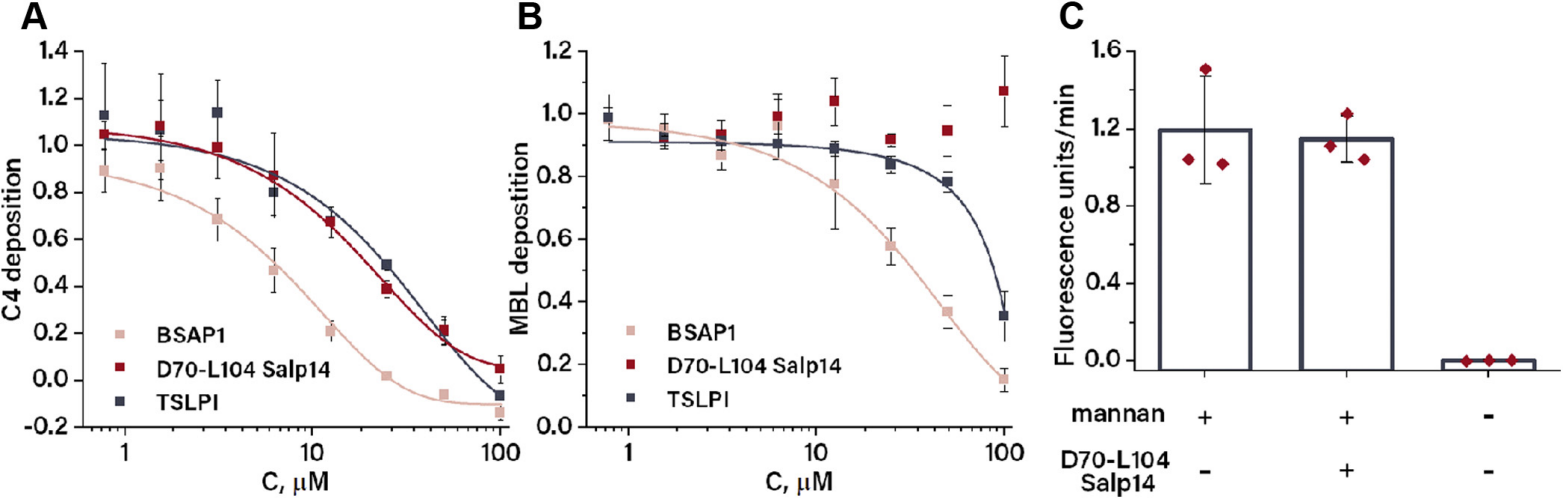
**Anti-complement activity of TSLPI, BSAP1, and D70-L104 Salp14.** Inhibition of lectin pathway activation by increasing concentrations of TSLPI, BSAP1, and D70-L104 Salp14 followed by C4b (*A*) or MBL (*B*) deposition. Each data point is averaged from three technical replicates and normalized to the buffer control in the absence of inhibitor. *C*, influence of D70-L104 Salp14 on the amidolytic activity of MBL-bound MASP1. Activity is followed by cleavage of VPR-ACM substrate and expressed as means of the slope of the fluorescence curve averaged from three measurements, and error bars indicate SD. BSAP1, BaSO_4_-adsorbing protein 1; MASP1, MBL-associated serine protease; MBL, mannan-binding lectin; TSLP, tick salivary lectin pathway; VPR-ACM, Boc-Val-Pro-Arg-7-amido-4-methylcoumarin.

The lectin pathway of the complement system is initiated by binding of pattern recognition proteins such as mannan-binding lectin (MBL) and ficolins to certain carbohydrates. MBL and ficolins circulate in blood in complex with MBL-associated serine proteases (MASPs), which upon binding to carbohydrate convert complement proteins C4 and C2, further unfolding the lectin pathway (19). As TSLPI is a known inhibitor of the lectin pathway, TSLPI/Salp14 proteins were tested in a lectin pathway activation ELISA assay using mannan activation and an anti-C4b antibody as readout. TSLPI blocked lectin pathway activation in a dose-dependent manner with an EC_50_ value around ∼25 μM (Fig. 4*A*). BSAP1 had a higher inhibitory activity with EC_50_ values of ∼8 μM, similarly to Salp14 and Salp9 (Fig. S3). As BSAP1 is a novel complement inhibitor, its influence on activation of the classical and alternative pathway has been assessed as well. BSAP1 at a concentration of 100 μM effectively inhibited activation of the classical pathway but had little effect on the alternative pathway (Fig. S4).

Surprisingly, D70-L104 Salp14 alone also effectively inhibited lectin pathway activation with an EC_50_ value of ∼17 μM. However, when a similar assay was performed using an anti-MBL antibody as readout, D70-L104 Salp14 had no effect on lectin pathway activation, whereas TSLPI and BSAP1 remained active with EC_50_ values of ∼90 and ∼26 μM, respectively (Fig. 4*B*). To assess the influence of D70-L104 Salp14 on MASPs, the amidolytic activity of MBL-bound MASP1 in the absence and presence of D70-L104 Salp14 was studied. To this end, active MBL–MASP complexes were captured from human serum using mannan-coated wells, and the amidolytic activity of MASP1 was assessed using a fluorogenic substrate. When serum was incubated in mannan-coated wells, the observed slope of the fluorescence signal was 1.2 ± 0.3 fluorescence units/min, whereas in the absence of mannan coating, no significant fluorescence signal was detected (Fig. 4*C*). Addition of 100 μM D70-L104 Salp14 to mannan-bound MBL–MASP complexes resulted in a slope of 1.1 ± 0.1 fluorescence units/min, which is comparable with control experiments in the absence of the peptide.

### NMR analysis and structure determination

To investigate a possible structure–activity relationship of TSLPI/Salp14 proteins, we attempted structure elucidation of TSLPI, BSAP1, and D70-L104 Salp14 using solution NMR spectroscopy. Although D70-L104 Salp14 embodies multiple lysines in virtually similar environments, all signals were unambiguously assigned using a series of ^15^N heteronuclear single quantum coherence (HSQC) and 2D decoupling in the presence of scalar interactions/NOESY spectra at different temperatures to remove severe signal overlap (Fig. S5). Nevertheless, the narrow chemical shift dispersion and the lack of long-range NOE signals indicated that the D70-L104 Salp14 C terminus adopts an unstructured random coil–like state.

**Figure 5:**
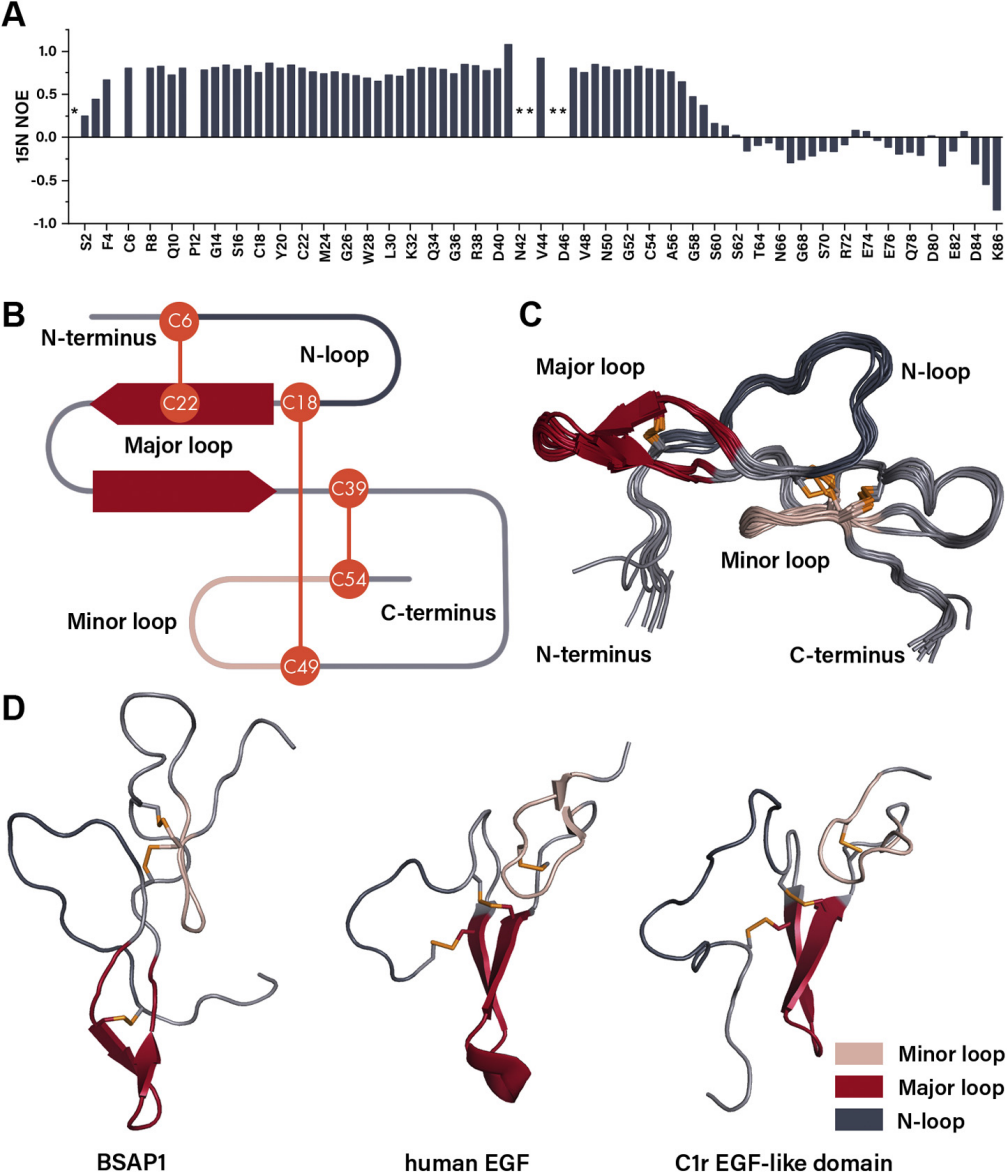
**The structure analysis of BSAP1.***A*, ^15^N heteronuclear NOE relaxation values of [^13^C, ^15^N] BSAP1 at pH 7.1 and 30 °C. Missing nonproline peaks are designated by an ∗. Schematic representation of the secondary structure and disulfide connectivity (*B*) and the superposition of the ten lowest-energy structures of BSAP1 (*C*). *D*, side-by-side comparison of BSAP1 structure with human EGF (Protein Data Bank ID: 1JL9) and C1r EGF-like domain (Protein Data Bank ID: 1APQ). Only the D1-S60 region of BSAP1 is shown for better visibility, disulfide bonds are shown in *orange*, structural elements are colored similarly in panels *B*–*D*. BSAP1; BaSO_4_-adsorbing protein 1; EGF, epidermal growth factor.

To allow profound structural analysis of TSLPI and BSAP1, both proteins were uniformly metabolically enriched with ^15^N and ^13^C and studied by 2D and 3D NMR spectroscopy. In the ^15^N HSQC spectrum of TSLPI, the vast majority of amide peaks showed severe resonance broadening, and a mere ∼30% of residues could be assigned (Fig. S6), irrespective of screening optimal conditions for spectra recording. This severely limited a detailed structure elucidation. In contrast, the ^15^N HSQC spectrum of BSAP1 showed nearly all amide signals with exceptions of D1, N42, A43, K45, and D46 (Fig. S7). ^15^N NOE relaxation data indicated that the structured region of the protein includes residues within a cysteine framework and spans roughly from S2 to S60 residues, whereas the C terminus remains flexible (Fig. 5*A*). Analysis of the 2D ^15^N NOESY spectrum yielded ∼1100 assigned NOE signals, of which 986 belonged to the D1-S60 region. Combined with TALOS-predicted dihedral angles and the previously determined disulfide bond network (C6–C22, C18–C49, and C39–C54) (15), these NOE signals were used as distant constraints for structure calculation using the Xplor-NIH software package (National Institutes of Health [NIH]; Table 1).

**Table 1:** Overview of constraints used for structure calculation and refinement statistics for the ten lowest-energy structures of BSAP1 obtained with Protein Structure Validation Server (38)

Summary of experimental constraints and structure validation	
Number of distance constraints	
Long [(i–j) > 5]	305
Medium [1 < (i–j) < 5]	63
Sequential [(i–j) = 1]	247
Intraresidual [i = j]	371
Total	986
Dihedral angle constraints	75
Average number of restrains per residue	17.7
Average atomic RMSD to the mean structure (Å)	
Backbone	0.7
Heavy atoms	1.0
Global quality scores (mean/Z score)	
Verify3D	0.19
Prosall	0.50
PROCHECK (φ–ψ)	−1.24
PROCHECK (all)	−1.09
MolProbity clash score	8.61
Ramachandran statistics (% of all residues)	
Most favored	62.2
Additionally allowed	35.1
Generously allowed	2.7
Disallowed regions	0.0

All values are given for the D1-S60 region of BSAP1.

The calculated BSAP1 structure could be divided into an N and C subdomain (Fig. 5, *B* and *C*). The N subdomain spans from D1 to F33 and includes the N loop framed by C6 and C18 and the major loop Y21-L30 with an antiparallel β sheet stabilized by four hydrogen bonds between residues M24-Q27 and C22-K29. The C subdomain spans roughly between C39 and S60 and is arranged by the C18-C49 and C39-C54 disulfide bonds. The region D46-S57 forms a tight minor loop maintained by hydrogen bonds between residues D46-S57, L53-C18, and C49-A37. Finally, the C subdomain is followed by a flexible and unstructured C-terminal region A61-K86.

## Discussion

Through millions of years of adaptation to a parasitic lifestyle, blood-sucking parasites have acquired numerous secreted bioactive compounds to counteract host hemostasis and immune responses. Ticks stand out from the rest of blood-feeding parasites because, due to active gene duplication, the tick's saliva composition is more complex and includes thousands of peptides and proteins (20, 21). Ticks target almost every aspect of their host defense mechanism, and therefore, tick salivary proteins are a rich source of candidates for the development of novel therapeutics and tools for surveying the underlying mechanisms of hemostasis and immune response. Here, we attempted a comprehensive functional and structural evaluation of proteins from the TSLPI/Salp14 family, which includes proteins with different reported activities—lectin pathway inhibition, plasminogen activation, and FXa inhibition (5, 9, 13). Moreover, we propose that the extrinsic blood coagulation pathway inhibitor BSAP1 (14) also belongs to the same protein family because of the similar cysteine pattern and a number of highly conserved residues along the amino acid sequence.

Published data over the anticoagulant activity of the proteins belonging to this family are ambiguous. Salp14 was described as an FXa inhibitor (13), but Ixonnexin, which shares 83% sequence identity with Salp14, has been found to increase the activity of FXa (5). Furthermore, BSAP1 has been reported to block the extrinsic pathway, but the nature of this inhibitory activity has not been revealed (14). To address this ambiguity, we tested several recombinantly expressed TSLPI/Salp14 proteins in the thrombin generation assay and found that only Salp14 significantly delayed thrombin generation in the micromolar concentration range. In fact, the positively charged C-terminal D70-L104 region of Salp14 alone was as active as the full-length protein, indicating that this region is responsible for the anticoagulant activity. This is in line with previous observations that Salp9Pac, which lacks a positively charged C terminus, shows no anticoagulant activity (13) and that Ixonnexin acts through interaction of polylysine stretches with plasminogen (5). Although D70-L104 Salp14 did not inhibit TF/FVIIa or FXa amidolytic activity, results of FV-inhibition assays indicated that it impairs FV activation and/or prothrombinase activity. The lysine-rich region ^89^TKKPKRKSKK^98^ of D70-L104 Salp14 seems to be pivotal for this activity, as its acetylation significantly impairs the inhibitory effect observed in the thrombin generation and FV(a) inhibition assays. It has been shown that the positive charge of polylysine chain alone is not sufficient to inhibit prothrombinase activity (22). Therefore, D70-L104 Salp14 most likely acts by mimicking the basic region of the TFPIα C terminus, which has been shown to interact with the exposed acidic region of the B domain of FV and to prevent the formation/activity of the prothrombinase complex (16). This interpretation is supported by the small increase in thrombin generation observed at the lowest concentrations of Salp14 D70-L104, probably reflecting the replacement of FV-bound TFPIα (strong prothrombinase inhibitor) by Salp14 D70-L104 (weaker prothrombinase inhibitor). The interaction of the TFPIα C terminus with the acidic region of FV also hinders the Arg^1545^ cleavage site, postponing full FV activation by FXa and thrombin (17), thus playing an important role in the early stages of the procoagulant response. The physiological relevance of such inhibition by Salp14 is in agreement with the fact that mRNA silencing of *salp14* and *salp9pac* genes caused significant reduction in the anticoagulant activity of tick salivary extracts (11). Interestingly, the activity of Salp14 resembles that of another salivary protein from *I. scapularis*—TIX-5, which specifically inhibits FV activation by FXa (23). Although both Salp14 and TIX-5 target the initiation phase of coagulation by impairing the formation of early prothrombinase, their mechanisms of action appear to be different. In fact, Salp14 is likely to bind to the acidic region of FV through its basic C terminus as the TFPIα C terminus, whereas TIX-5 has been shown to interact with a different site of the FV B domain as well as with phospholipids (24).

D70-L104 Salp14 also prevents the activation of the lectin pathway of the complement system by inhibiting C4 deposition, without blocking MBL binding to mannan or the amidolytic activity of MASP1. MASPs are multidomain proteins that embody CUB1-EGF-CUB2-CCP1-CCP2-SP domains (24). CUB1-EGF-CUB2 domains are involved in MASP dimerization and its binding to collagen domains of MBL. It has been demonstrated that lysine can inhibit lectin pathway activation at micromolar concentrations and cause dissociation of the MBL–MASP-1 complex through interaction with a lysine-binding pocket of CUB2 (25). Taking into account that D70-L104 Salp14 is unstructured, it could block the lectin pathway in a similar way through its lysine-rich regions. Interestingly, the Kunitz-2 domain of TFPIα has been shown to inhibit the amidolytic activity of MASP-2, whereas the Kunitz-3 domain and the C terminus partially inhibit lectin pathway activation but do not affect the amidolytic activity of MASP-2 (26). The data presented here provide a possible explanation for the TFPIα C terminus activity in the lectin pathway without interfering with the MASP-2 proteolytic activity, similar to D70-L104 Salp14.

**Figure 6:**
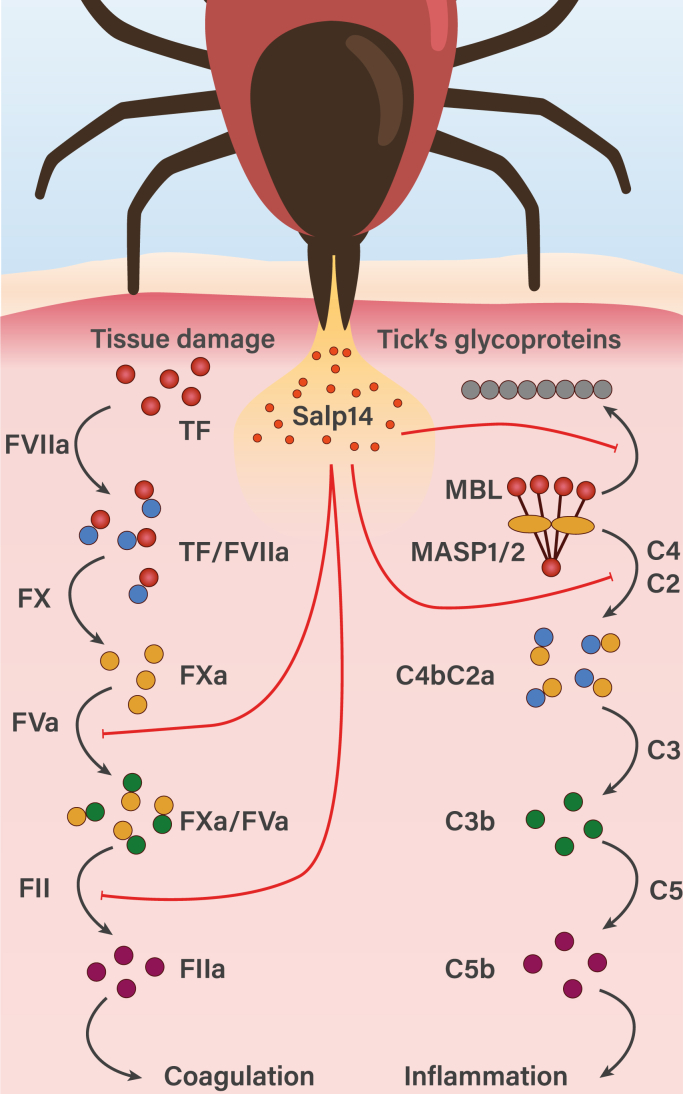
**Schematic representation of the extrinsic coagulation and lectin complement pathways and Salp14 inhibitory activity**.

All tested proteins of the TSPLI–Salp14 family inhibited activation of the lectin pathway of the complement system. BSAP1 not only prevents MBL binding to mannan similarly to TSLPI but also blocks the classical pathway as well. Therefore, one may suggest that TSLPI–Salp14 proteins are primarily complement inhibitors, whereas the secondary anticoagulant activity is acquired by some proteins, like Salp14, through mutations of the C-terminal region (27). During a blood meal, the tick's mouthpart penetrates the host's skin, damaging small blood vessels. This simultaneously triggers the extrinsic coagulation pathway by exposing TF from damaged tissue and the lectin complement pathway because of the interaction of circulating MBL with the pathogen's glycoproteins. Salp14 inhibits both the extrinsic pathway and the lectin complement pathway at early stages (Fig. 6), whereas other proteins of the TSLPI–Salp14 family would only block the latter.

Although TSLPI–Salp14 proteins seem to bind directly to MBL, the binding mode and structural determinants of MBL binding remain elusive, because of the intricate structure of MBL and MBL–MASP complexes. As a stepping stone to this endeavor, we solved the structure of BSAP1 using solution NMR spectroscopy. Although BSAP1 shares no sequence homology and disulfide connectivity with EGF-like domain proteins, it appears to have the same spatial topology as EGF-like domain proteins (28, 29). These proteins could be similarly divided into an N subdomain with a β sheet in a major loop and the N terminus attached to it by a disulfide bond, and a C subdomain that embodies a minor loop (Fig. 5*D*). Therefore, BSAP1 could be structurally attributed to noncanonical EGF-like domain proteins. Although EGF-like domains are ubiquitous among the animal kingdom, including ticks (30, 31), BSAP1 is to the best of our knowledge the first example of an EGF-like domain protein found among tick salivary proteins.

In summary, in the present study, proteins from the TSLPI–Salp14 family were functionally and structurally characterized. Our data indicate that these proteins are primarily lectin pathway inhibitors. Only Salp14 also exerts anticoagulant activity because of the polylysine motif in its tail, which prevents the formation of the prothrombinase complex similarly to the C terminus of TFPIα. Structure elucidation showed that BSAP1 adopts a novel noncanonical EGF-like domain fold, but its structure–function relationship remains to be unraveled.

## Experimental procedures

### Protein expression, refolding, and purification

Vectors containing the complementary DNAs of the proteins of interest (Table S1) were purchased from GenScript. Tick proteins were expressed in *E. coli*, refolded and purified as described previously (32), with minor adaptations in the cell lysis procedure. Namely, for lysis, bacterial pellets were resuspended in 6 M Gdn–HCl, 50 mM Tris, and pH 8 at a concentration of 1 mg/ml and stirred for 1 h at RT. Then cell debris was removed by centrifugation at 10,000 rpm for 20 min at 4 °C, and the soluble fraction was dialyzed overnight against 0.5% acetic acid using a 3.5 kDa Spectra/Por RC membrane (Repligen). After dialysis, the soluble fraction was separated by centrifugation at 10,000 rpm for 20 min at 4 °C and lyophilized.

### Peptide synthesis

Peptides (Table S2) were synthesized using standard Boc-based SPPS (15) on Boc-Leu-PAM resin. For site-selective acetylation, corresponding lysines were incorporated in a peptide chain using Boc-Lys(Fmoc)-COOH building blocks. After chain assembly, Fmoc protecting groups were removed by 20% piperidine, and the resin was treated with 0.25 M acetic anhydride and 0.25 M piperidine in *N*,*N*-dimethylformamide two times for 2 min.

### NMR spectroscopy and structure calculation

NMR samples were prepared, and NMR spectra were recorded using a Bruker Avance III HD 700 MHz spectrometer, equipped with a cryogenically cooled TCI probe as described previously (32). Detailed description of procedures, the list of recorded spectra, and their conditions can be found in the Supporting information. Spectra processing was performed by Topspin 3.2 (Bruker) and NMRFAM-Sparky 1.470 software (33). Dihedral angles were predicted using TALOS+ Web server (34). Structure calculations were performed with Xplor-NIH software (35, 36) using the EEFx (effective energy function) force field (37). In short, 100 initial structures were calculated with the fold protocol, then the ten lowest-energy structures were selected, and another 100 structures were calculated using the refinement protocol. The final ensemble of ten lowest-energy structures was analyzed using the Protein Structure Validation Server (38).

### Complement activation assays

Lectin pathway complement activation in human serum was assessed using an ELISA-based assay as described previously (9) with minor alterations. In short, Nunc Maxisorb flat-bottom 96-well plates (Thermo Fisher Scientific) were coated with 100 μl of 10 μg/ml mannan in 15 mM Na_2_CO_3_, 35 mM NaHCO_3_, 5 mM NaN_3_, and pH 9.6. After overnight incubation at 4 °C, wells were blocked with 100 μl of 10 mM Tris, 145 mM NaCl, 15 mM NaN_3_, 1 mg/ml bovine serum albumin (BSA), pH 7.4 for 2 h at RT. Then, wells were washed three times with 100 μl of 10 mM Tris, 145 mM NaCl, 5 mM CaCl_2_, 0.05% Tween 20, and pH 7.4. Pooled normal human serum was diluted 1:125 in 5 mM barbital, 145 mM NaCl, 1 mM MgCl_2_, 2 mM CaCl_2_, 0.02% Tween-20, and 1 mg/ml BSA containing the desired concentration of the protein of interest and incubated in the wells for 1 h at 37 °C. Subsequently, wells were washed and incubated with biotinylated antihuman C4b (Novus Biologicals) or MBL antibody (Sino Biological) for 45 min at RT. Then, streptavidin-conjugated horseradish peroxidase in washing buffer was added to the wells and incubated for 30 min at RT. After washing, 3,3′,5,5′-tetramethylbenzidine substrate was added, and the absorbance at 450 and 630 nm was measured using a plate reader.

Activation of the classical and alternative pathways has been assessed using WIESLAB Complement System Classical and Alternative Pathway Kits (Svar), respectively, according to the manufacturer's protocol. Reconstituted human serum provided with the kits was used for inhibition activity tests.

### Amidolytic activity of MASP1

The activity of MBL-bound MASP1 was measured as described (39). In short, Nunc Maxisorb flat-bottom 96-well plates (Thermo Fisher Scientific) were coated with 1 mg/ml mannan and blocked as described previously. Then 50 μl of normal human serum (Sigma–Aldrich) were diluted 1:1 in 20 mM Hepes, 2 M NaCl, 10 mM CaCl_2_, pH 7.4 and incubated at 4 °C for 1 h. After incubation, wells were washed two times with 100 μl of 10 mM Hepes, 1 M NaCl, 5 mM CaCl_2_, 0.05% Tween-20, pH 7.4, and another three times with 100 μl of 10 mM Tris, 140 mM NaCl, 5 mM CaCl_2_, and 0.05% Tween-20. Finally, 200 μl of 20 mM Hepes, 5 mM CaCl_2_, 0.1 mM Boc-Val-Pro-Arg-7-amido-4-methylcoumarin, pH 8.5 with or without D70-L104 Salp14 was added, and the fluorescent signal (λ_ex_ = 390 nm, λ_em_ = 460 nm) was detected using a Fluoroskan Ascent reader (Thermo Labsystems)

### Thrombin generation

Thrombin generation was measured by calibrated automated thrombinography (40), essentially as described before (17). Briefly, coagulation was initiated in normal pooled plasma with 2 pM TF (Dade Innovin), 30 μM phospholipid vesicles (1,2-dioleoyl-*sn*-glycero-3-phosphoserine/1,2-dioleoyl-*sn*-glycero-3-phosphocholine/1,2-dioleoyl-*sn*-glycero-3-phosphoethanolamine in 20/60/20 M ratio) and 16 mM CaCl_2_, in the presence of 40 μg/ml thermostable inhibitor of contact activation and 0 to 20 μM tick protein. Thrombin activity was followed using fluorogenic substrate Z-Gly-Gly-Arg-AMC (I-1140; Bachem), which was added to the plasma together with the CaCl_2_ solution. Fluorescence was read in a Fluoroskan Ascent reader (Thermo Labsystems), and thrombin generation curves were calculated with the Thrombinoscope software (Thrombinoscope). Lag time and peak height were used as the main read-out parameters. Control experiments indicated that none of the tick proteins affected the slope of the thrombin calibrator used to calibrate the assay.

### Chromogenic assays for FVIIa and FXa

About 0.25 nM FVIIa (NovoSeven; Novo Nordisk) was preincubated with 5 nM TF (Dade Innovin; Siemens) in HN-BSA buffer (20 mM Hepes, 140 mM NaCl, 5 mg/ml BSA, pH 7.4) supplemented with 3 mM CaCl_2_ for 15 min at 37 °C to allow the formation of the TF–FVIIa complex. After incubating the complex with 0 to 20 μM tick protein for another 15 min at 37 °C, the residual FVIIa activity was probed by adding 0.5 mM Spectrozyme FVIIa (ImmBioMed) and following the absorbance at 405 nm in a plate reader. 0.1 nM human FXa (Enzyme Research Laboratories) in HN-BSA buffer was incubated with 0 to 20 μM tick protein for 15 min at 37 °C. Residual FXa activity was probed by adding 120 μM Biophen CS-11 (65) (Aniara) and following the absorbance at 405 nm.

### FV inhibition assay

The inhibition of FV activation and/or prothrombinase activity by tick proteins was determined using a previously described assay developed to probe the susceptibility of FV(a) to inhibition by the TFPIα C terminus (18). Briefly, FV in 1/1000 diluted normal human plasma was activated with a suboptimal FXa concentration (20 pM) in the presence of 30 μM phospholipid vesicles (1,2-dioleoyl-*sn*-glycero-3-phosphoserine/1,2-dioleoyl-*sn*-glycero-3-phosphocholine/1,2-dioleoyl-*sn*-glycero-3-phosphoethanolamine in 20/60/20 M ratio) and 100 nM tick peptide for 3 min at 37 °C, and prothrombinase activity on exogenously added prothrombin (0.5 μM) was followed continuously for at least 30 min using chromogenic substrate S-2238 (Chromogenix; 0.5 mM final concentration). Each parabolic absorbance tracing was fitted to a second-order polynomial equation, and the prothrombinase rate (thrombin/min/FXa) was calculated from the coefficient of the second order term, as described (18).

### Bioinformatics

For homology search, the BLASTp algorithm against UniProtKB database was used (41). Annotated signal peptides were removed manually. Multiple sequence alignment was performed using Clustal Omega algorithm (42) with default settings and visualized by JalView 2.10 software (43).

## Data availability

The structure of BSAP1 presented in this article has been deposited in the Protein Data Bank under the accession code 7NE8.

## Supporting information

This article contains supporting information.

## Conflict of interest

The authors declare that they have no conflicts of interest with the contents of this article.
