# Supplementary material for: Molecular basis of anticoagulant and anticomplement activity of the tick salivary protein Salp14 and its homologs
Source: J Biol Chem. 2021 Jun 9;297(1):100865. doi: 10.1016/j.jbc.2021.100865 (PMC8294578; doi:10.1016/j.jbc.2021.100865)
Supplement: Supplemental Figures S1–S7 and Tables S1–S2 [file mmc1.pdf]

Molecular basis of anticoagulant and anti-complement activity of the tick salivary protein Salp14 and its homologues

Stepan S. Denisov, Johannes H. Ippel, Elisabetta Castoldi, Tilman M. Hackeng, Ingrid Dijkgraaf*

Department of Biochemistry, University of Maastricht, Cardiovascular Research Institute Maastricht (CARIM), Universiteitssingel 50, 6229 ER, Maastricht, The Netherlands.

*Corresponding author: Ingrid Dijkgraaf

E–mail: i.dijkgraaf@maastrichtuniversity.nl
phone: +31-43-3881681
fax: +31-43-3884159

Running title: Dual inhibitory activity of the basic tail of Salp14

Keywords: coagulation factor; complement system; nuclear magnetic resonance (NMR); protein structure; parasite; ticks; lectin pathway; anticoagulants;

Contents

Supplemental methods 2

NMR experiments 2

Table S1. ORF sequences used for protein expression 3

Table S2. Sequences of synthetic peptides 3

Figure S1. HPLC traces of folding (left) and purified proteins 4

Figure S2. HPLC traces of purified synthetic peptides 5

Figure S3. Inhibition of lectin pathway activation by increasing concentrations of Salp9 and Salp14 .. 6

Figure S4. Activation of alternative and classical complement pathways in the presence of different concentrations of BSAP1 7

Figure S5. ^{15}N-^{1}H HSQC amide spectrum of 870 µM D70-L104 Salp14 8

Figure S6. ^{15}N-^{1}H HSQC spectrum of 150 µM [^{13}C, ^{15}N] TSLPI at pH 4.8, 37°C 9

Figure S7. ^{15}N-^{1}H HSQC spectrum of 320 µM [^{13}C, ^{15}N] BSAP1 at pH 7.1, 30°C. 10

Supplemental methods
NMR experiments.

NMR sample preparation BSAP1

NMR samples of E.coli expressed Met0-BSAP1 were made as 0.30 to 0.35 mM solutions in KPi 25 mM buffer (pH 7.1), 0.1 mM EDTA, 0.2 mM sodium azide, containing 1% (v/v) D_2O and 2 μM DSS-d^6 as an internal chemical shift reference. Addition of 5 mM $CaCl_2$ did not change the NMR spectrum, but was used for labelled BSAP-1 samples to ensure that enough calcium is present, because it was reported in literature that BSAP1 is a possible calcium binding protein. Samples of uniformly [^{13}C,^{15}N] labelled BSAP1 were prepared at 0.32 mM concentration in 160 μl volume 3 mm NMR tubes, after extensive ultracentrifugation of the protein in phosphate buffer over Amicon 3K filter devices for buffer exchange.

NMR sample preparation TSLPI

TSLPI NMR samples were prepared at pH 4.8 in deuterated sodium acetate-d^3 25 mM buffer and at pH 7.15 in KPi 25 mM buffer, with added 0.1 mM EDTA and 0.2 mM azide, 2 μM DSS-d^6 and 1 % (v/v) D_2O. In both cases, HSQC ^{15}N-^1H spectra indicate folded protein, but rather show a limited number of visible amide peaks, with most resonances severely broadened or missing. However, optimal sample conditions for data collection on uniformly [^{13}C,^{15}N] labelled Met0-TSLPI were decided at 25°C at pH 7.15 and 37°C at pH 4.8, all spectra recorded at a protein concentration of 0.15 mM measured in 3 mm NMR tubes.

NMR sample preparation of the synthetic Salp14 D70-L104 peptide

Samples of Salp14 D70-L104 made by dissolving freeze-dried powder in 25 mM sodium acetate-d^3 buffer (pH 4.45), 0.1 mM EDTA, 0.2 mM azide, 2 μM DSS-d^6 and 1 to 2 % (v/v) D_2O. Final peptide concentrations were 0.87 mM in a volume of 560 μl in a 5 mm NMR tube. Spectra for the sequential assignment were recorded at 25°C, a temperature which is low enough to ensure the observation of exchangeable arginine HN sidechain resonances in proton correlated spectra.

NMR spectroscopy

NMR spectra were recorded on a Bruker (Rheinstetten, Germany) Avance III HD 700 MHz NMR spectrometer, equipped with a cryogenically-cooled [^{15}N,^{13}C,^1H] TCI probe. Sequential assignment for each non-labelled Salp14 peptides were carried out on a series of 2D spectra, namely NOESY (250 ms mixing time) and DIPSI (80 ms mixing time), supported by natural abundance HSQC ^{15}N-^1H, HSQC ^{13}C-^1H, and DIPSI-HSQC ^{13}C-^1H (70 ms mixing time) spectra.

For the double labelled BSAP-1 and TSLPI proteins, the following triple resonance 3D spectra were recorded to aid the sequential assignment: HNCO, HNcaCO, CBCAcoNH, HNCACB, b_HNCACB (Asn/Gln amino), HBHAcoNH, HNCA, HNcoCA (TSLPI only), and HCCh-DIPSI (13 msec mixing time). In addition to its reference 2D HSQC ^{15}N-^1H and alifatic and aromatic optimized HSQC ^{13}C-^1H constant time spectra, ^{13}C-detected 2D C_CON, and HBCBcgcdHD, HBCBcgcdceHE were recorded to complete assignments. Finally, ^{15}N-^1H CLEANEX spectra, to pinpoint solvent-exposed amide and amino protons, together with heteronuclear ^{15}N-NOE spectra were recorded, to probe the intrinsic conformational flexibility of the protein backbone. For structural analysis of the proteins, high resolution 2D NOESY (150 ms mixing time), 3D ^{15}N-edited NOESY (150 ms mixing time BSAP-1 and 125 ms mixing time TSLPI), and 3D ^{13}C-edited-NOESY (TSLPI only, 150 ms mixing time) were acquired. Pulse sequences used for these NMR experiments were taken from the standard Bruker pulse sequence library.

Table S1. ORF sequences used for protein expression

Vector name	ORF
BSAP1<br>pET23a<br>*NdeI-EcoRI*	cat atg gac agc gag ttc ccg tgc ccg cgt aag cag caa ccg gcg ggc aac agc gag tgc<br>H M D S E F P C P R K Q Q P A G N S E C<br>agc tac tat tgc gaa atg aac ggc cag tgg aag ctg ggc aaa ttt caa aac ggt gcg cgt<br>S Y Y C E M N G Q W K L G K F Q N G A R<br>tgc gac tac aac gcg gtg aaa gat ggc gtt tgc aac gag ggt ctg tgc tat gcg agc ggc<br>C D Y N A V K D G V C N E G L C Y A S G<br>gat agc gcg agc aac acc cag aac caa ggt ggc agc cgt cgt cag gag aac gaa gac caa<br>D S A S N T Q N Q G G S R R Q E N E D Q<br>ggt gac gat gaa tgg gat cgt aag taa gaa ttc<br>G D D E W D R K - E F
TSLPI<br>pET30a<br>*NdeI-HindIII*	cat atg cat aac tgc cag aac ggc acc cgt ccg gcg agc gaa gag aag cgt gaa ggc tgc<br>H M H N C Q N G T R P A S E E K R E G C<br>gac tac tac tgc tgg aat gcg gag acc aat agc tgg gac aag ttc ttt ttc ggt aac ggc<br>D Y Y C W N A E T N S W D K F F F G N G<br>gag cgt tgc ttt tac aac gat ggt ggc gaa ggt ctg tgc caa aac ggc gaa tgc cac ctg<br>E R C F Y N D G G E G L C Q N G E C H L<br>acc acc gat agc agc gtt ccg aat gat agc gat gtg taa aag ctt<br>T T D S S V P N D S D V - K L
Salp9<br>pET23a<br>*NdeI- EcoRI*	cat atg cac gac tgc caa aac ggt acc cgt ccg gcg agc gag gaa aag cgt gaa ggc tgc<br>H M H D C Q N G T R P A S E E K R E G C<br>gac tac tat tgc tgg aac acc gaa acc aag agc tgg gat aaa ttc ttt ttc ggt aac ggc<br>D Y Y C W N T E T K S W D K F F F G N G<br>gaa cgt tgc ttt tac aac aac ggt gac gaa ggc ctg tgc caa aac ggc gag tgc cac ctg<br>E R C F Y N N G D E G L C Q N G E C H L<br>acc acc gat agc ggc gtg ccg aac gac acc gat gcg aaa atc gag gaa acc gag gaa gag<br>T T D S G V P N D T D A K I E E T E E E<br>ctg gag gcg taa gaa ttc<br>L E A - E F
Salp14<br>pET23a<br>*NdeI- EcoRI*	cat atg cac aac tgc caa aac ggt acc cgt ccg gcg agc gag caa gac cgt gaa ggc tgc<br>H M H N C Q N G T R P A S E Q D R E G C<br>gat tac tat tgc tgg aac gcg gaa acc aag agc tgg gac cag ttc ttt ttc ggt aac ggc<br>D Y Y C W N A E T K S W D Q F F F G N G<br>gaa aaa tgc ttt tac aac agc ggt gat cac ggc acc tgc caa aac ggc gag tgc cac ctg<br>E K C F Y N S G D H G T C Q N G E C H L<br>acc aac aac agc ggt ggc ccg aac gaa acc gac gat tat acc ccg gcg ccg acc gaa aag<br>T N N S G G P N E T D D Y T P A P T E K<br>ccg aaa cag aag aaa aag aaa acc aag aaa acc aag aaa ccg aag cgt aaa agc aag aaa<br>P K Q K K K K T K K T K K P K R K S K K<br>gac caa gaa aag aac ctg taa gaa ttc<br>D Q E K N L - E F

Table S2. Sequences of synthetic peptides

Name	Sequence
**D70-L104 Salp14**	NH_2-DYTPAPTEKPKQKKKKTKKTKKPKRKSKKDQEKNL-COOH
**D70-L104 acSalp14**	NH_2-<br>DYTPAPTEKPKQKKKKTKKTK(ac)K(ac)PK(ac)RK(ac)SK(ac)K(ac)DQEKNL-<br>COOH
**D70-L104 Salp14/TFPI**	NH_2-DYTPAPTEKPKQKKKKTKK**LIKTKRKRKK**DQEKNL-COOH
**TFPI Cterm**	acetyl-GFIQRISKGGLIKTKRKRKKQRVKIAYEEIFVKNM-COOH

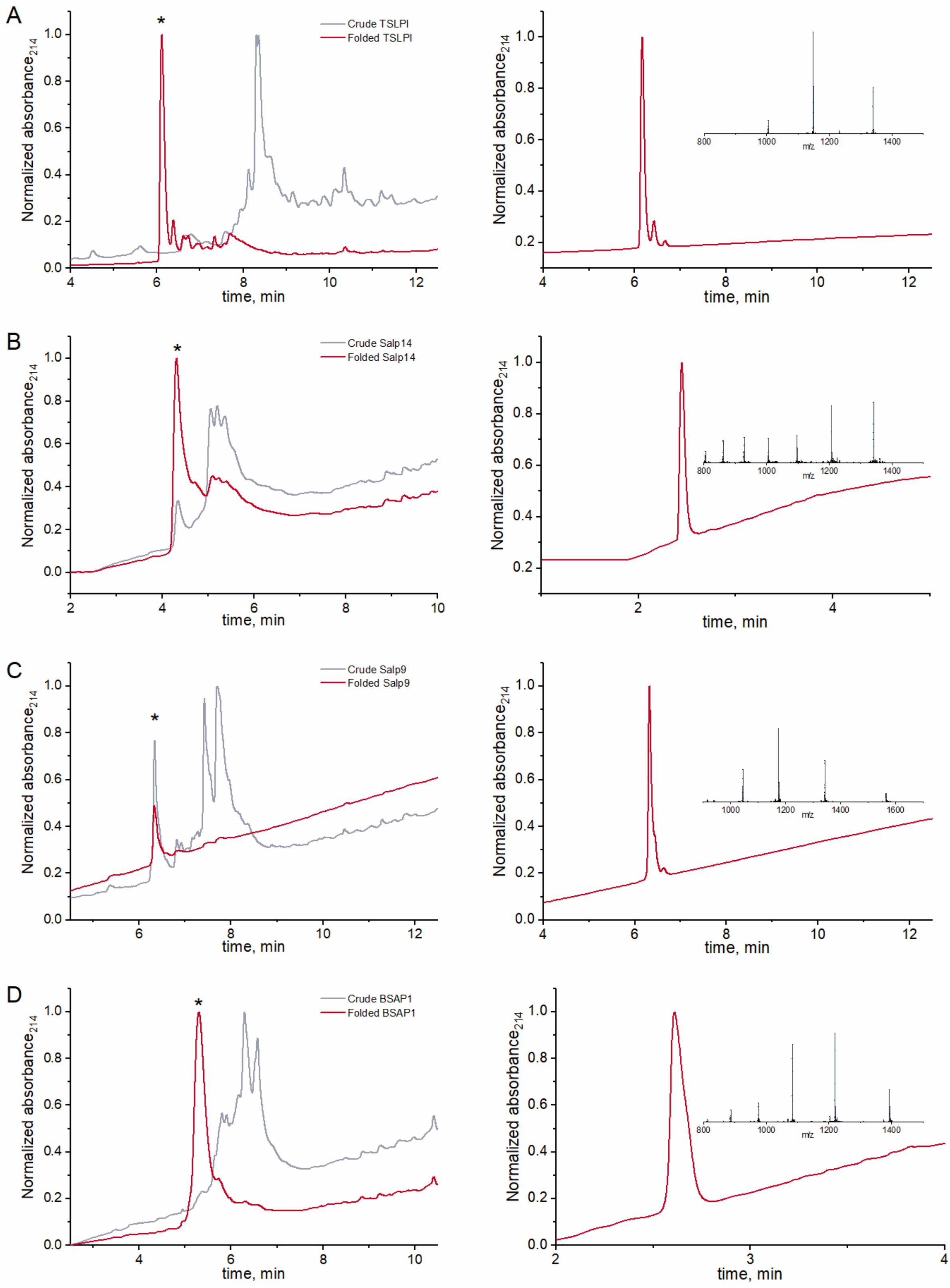

Figure S1. HPLC traces of folding (left) and purified proteins (right) for TSLPI (**A**), Salp14 (**B**), Salp9 (**C**), and BSAP1 (**D**). MS spectra of proteins are shown as inserts, * indicates the correct folded protein. Calculated M^+ and observed $[MH]^+$ masses for folded proteins are: TSLPI – calculated 8027.2 Da, observed 8028.3 Da; Salp14 – calculated 12056.5 Da, observed 12057.0 Da; Salp9 – calculated 9385.9 Da, observed 9386.6 Da; BSAP1 – calculated 9743.1, observed 9744.27 Da.

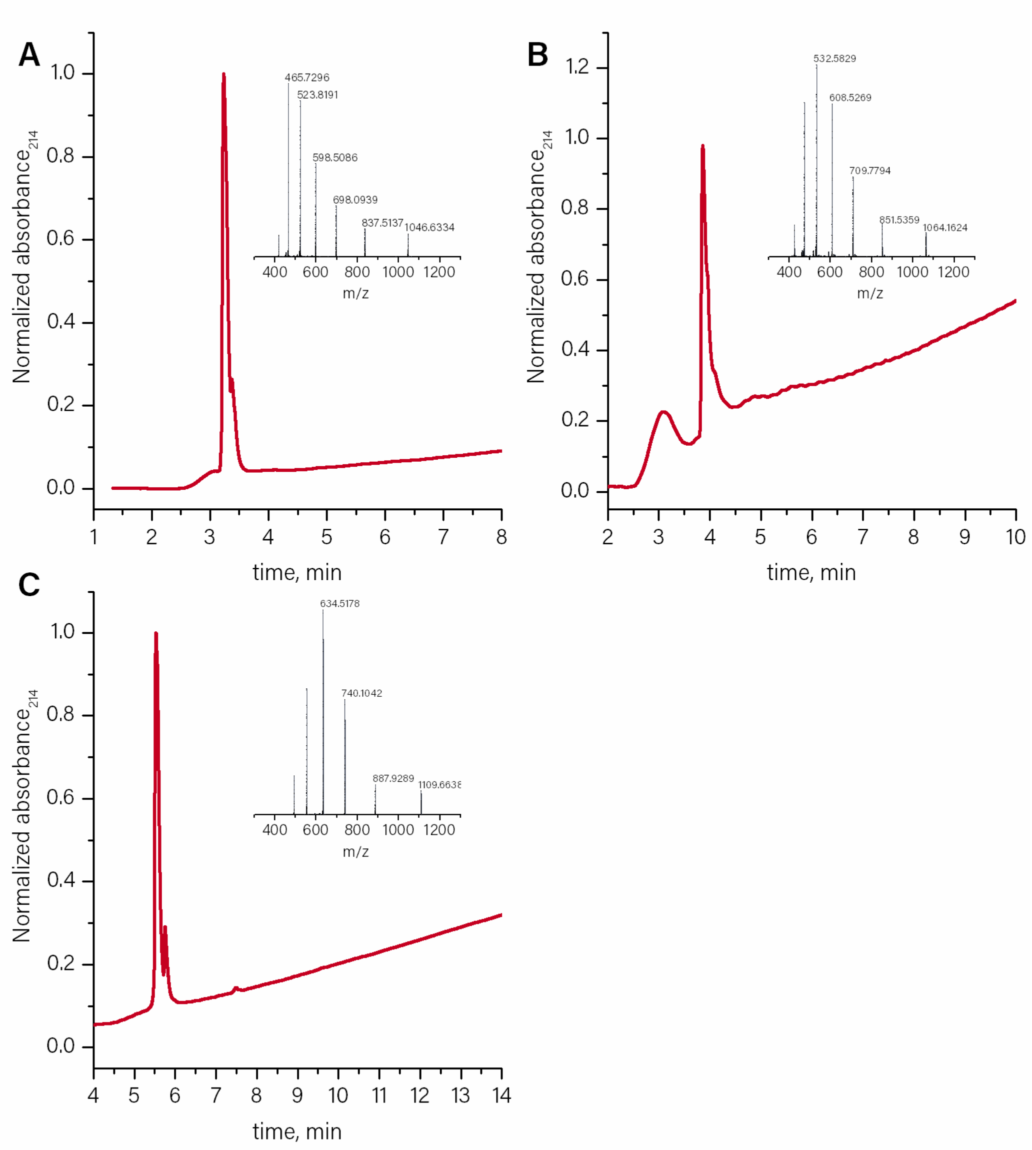

Figure S2. HPLC traces of purified synthetic peptides: A - D70-L104 Salp14; B - D70-L104 acSalp14; C - D70-L104 Salp14/TFPI. MS spectra are shown in inserts. Calculated M^+ and observed $[MH]^+$ masses are: D70-L104 Salp14 – calculated 4180.4 Da, observed 4181.5 Da; D70-L104 acSalp14 – calculated 4432.6 Da, observed 4433.6 Da; D70-L104 Salp14/TFPI – calculated 4250.5 Da, observed 4251.6 Da.

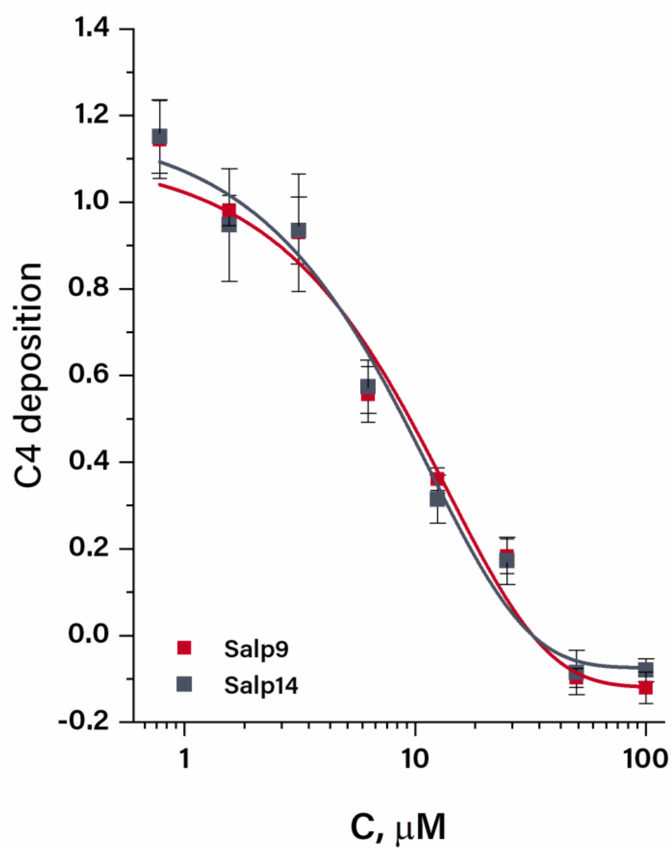

Figure S3. Inhibition of lectin pathway activation by increasing concentrations of Salp9 and Salp14 followed by C4b deposition. Each data point is averaged from three technical replicates and normalized to the buffer control in the absence of inhibitor.

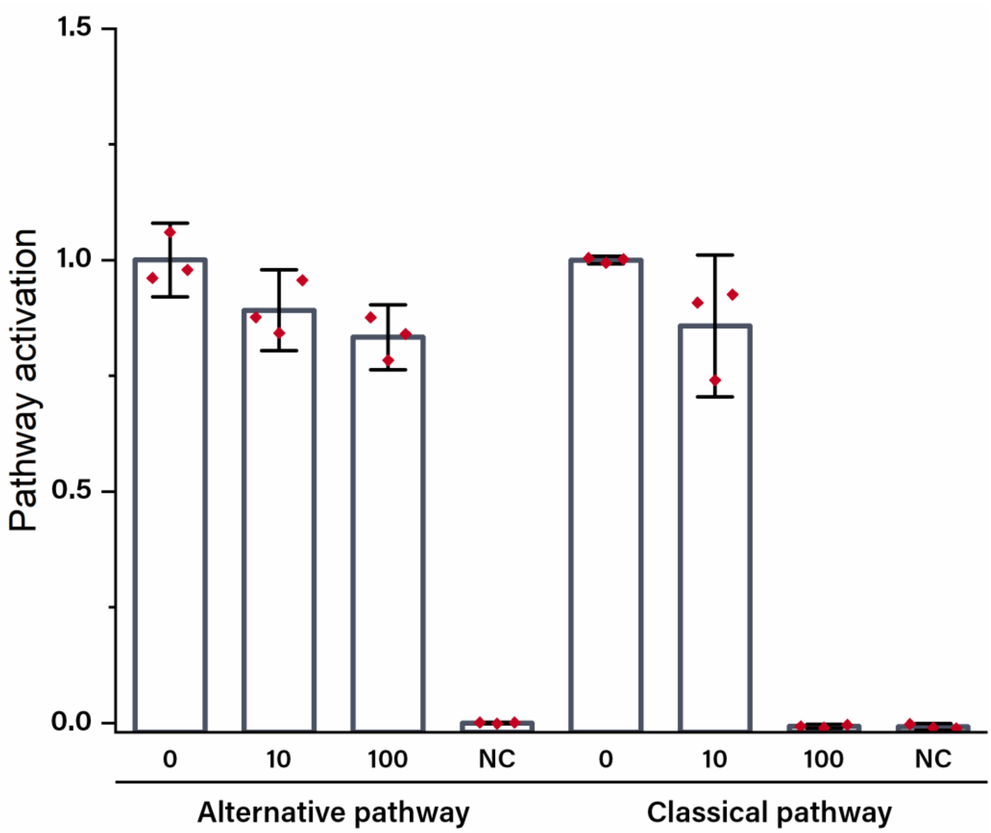

Figure S4. Activation of alternative and classical complement pathways in the presence of different concentrations of BSAP1. Each data point is normalized to the mean value of the positive control in the absence of the inhibitor. Bars represent mean values and error bars SD. NC stands for negative control serum. Concentrations are indicated in µM.

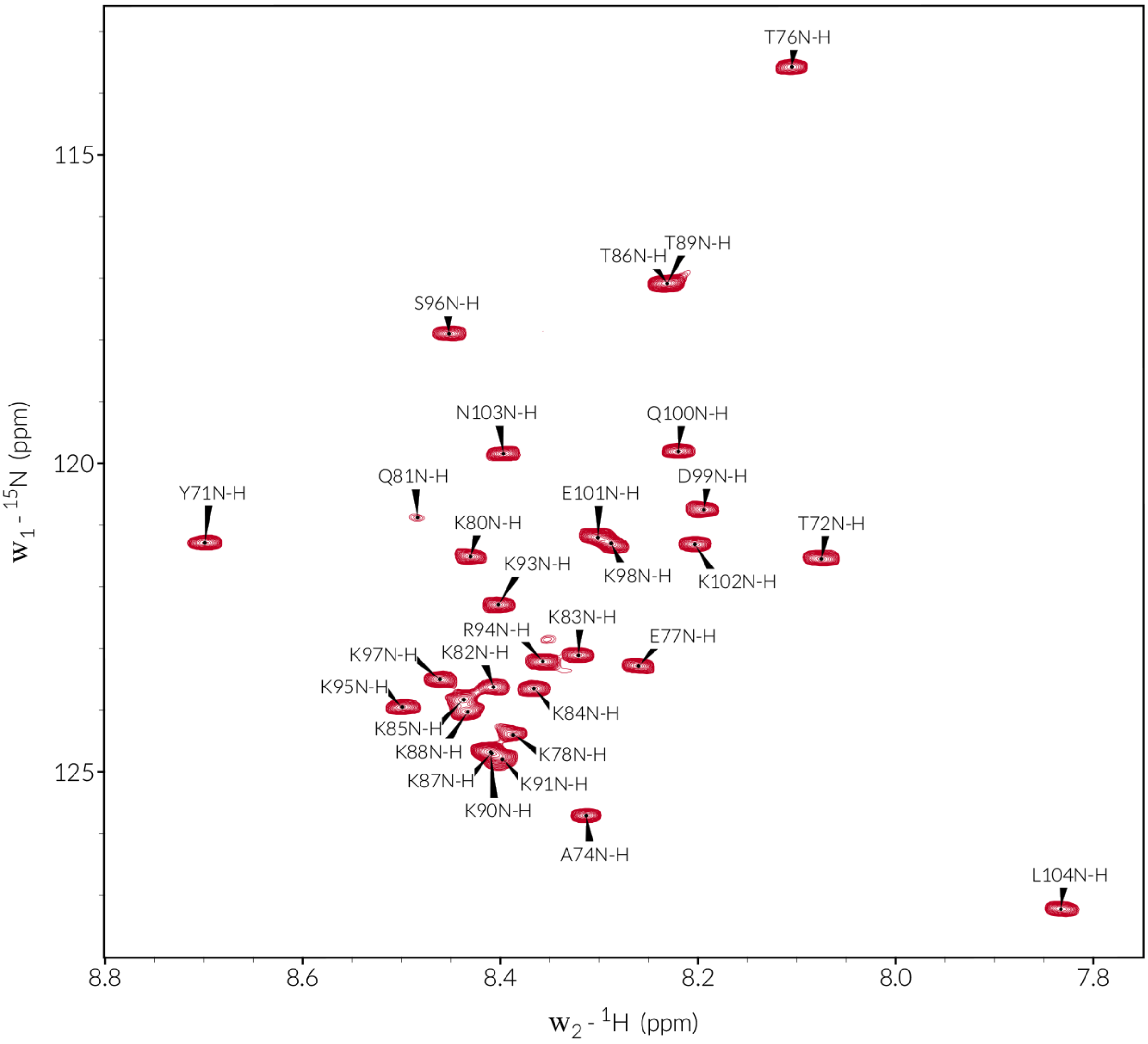

Figure S5. ^{15}N-^{1}H HSQC amide spectrum of 870 µM D70-L104 Salp14 at pH 4.5, 30°C.

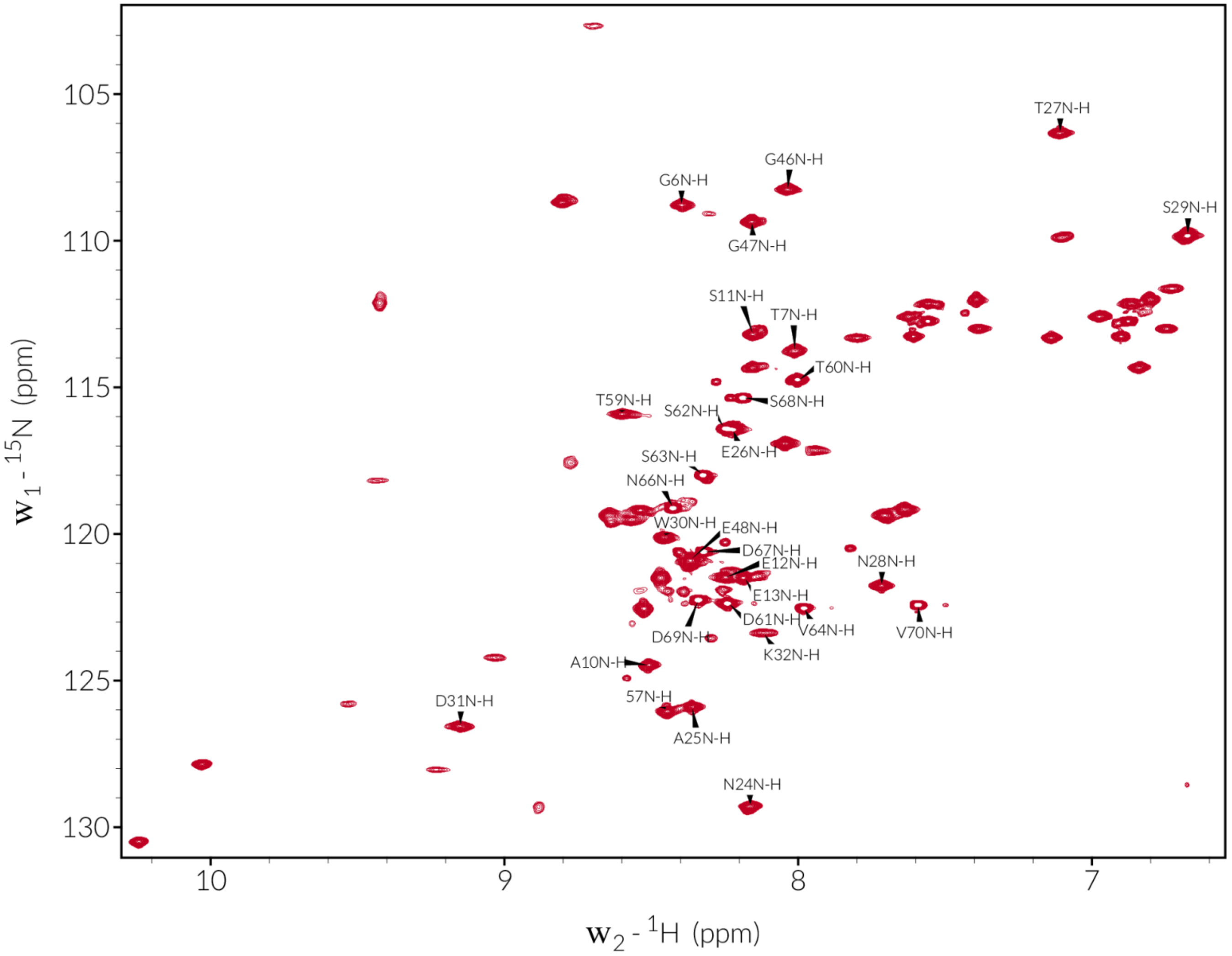

Figure S6. ^{15}N-^{1}H HSQC spectrum of 150 µM [^{13}C, ^{15}N] TSLPI at pH 4.8, 37°C. Assignments of tryptophan, asparagine, glutamine, and arginine side chain peaks are left out for improved visibility.

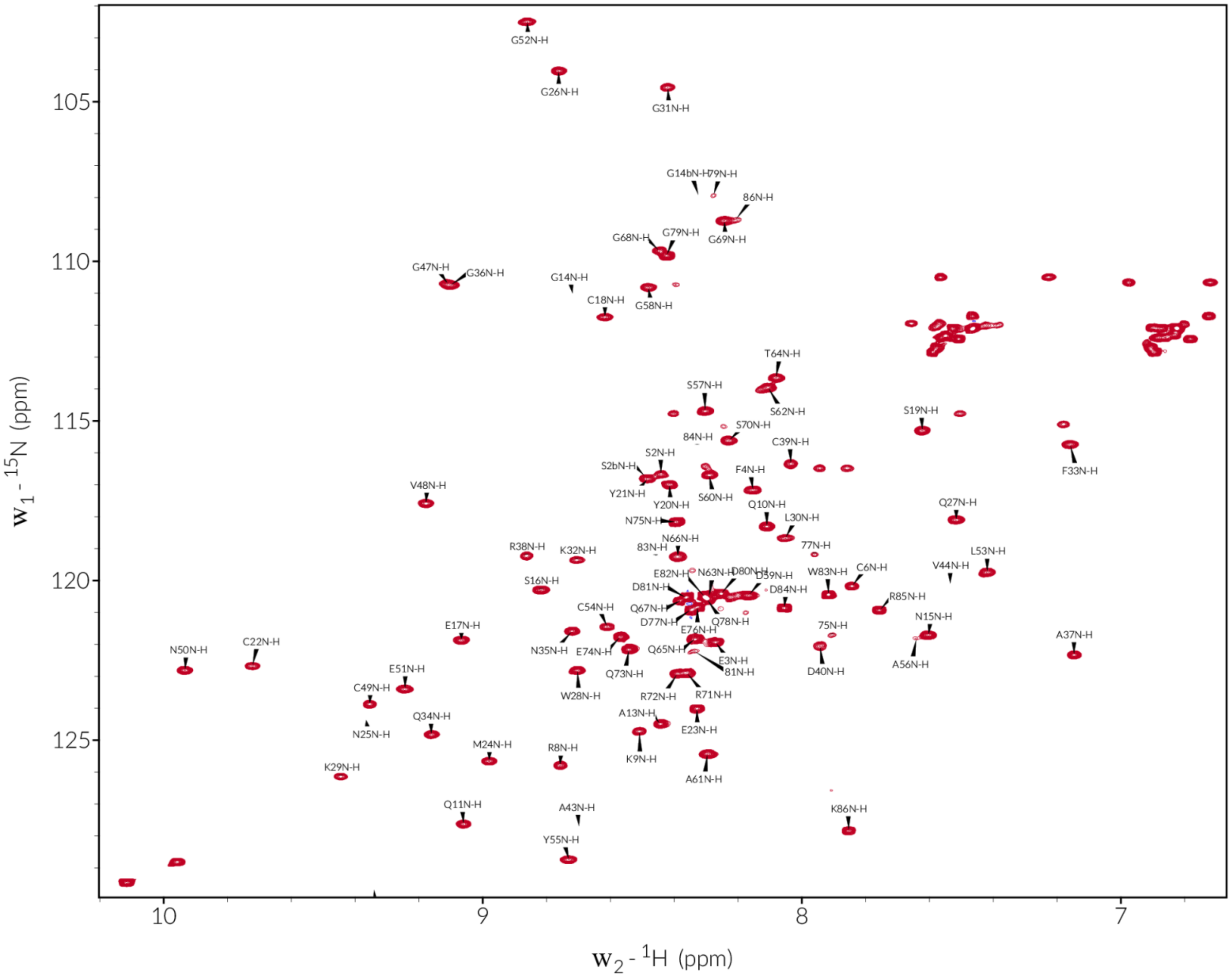

Figure S7. ^{15}N-^{1}H HSQC spectrum of 320 µM [^{13}C, ^{15}N] BSAP1 at pH 7.1, 30°C. Assignments of tryptophan, asparagine, glutamine, and arginine side chain peaks are left out for improved visibility.
